# Testing the “read-across hypothesis” by investigating the effects of ibuprofen on fish

**DOI:** 10.1016/j.chemosphere.2016.08.041

**Published:** 2016-11

**Authors:** Alpa Patel, Grace H. Panter, Henry T. Trollope, Yohanna C. Glennon, Stewart F. Owen, John P. Sumpter, Mariann Rand-Weaver

**Affiliations:** a Biosciences, Institute of Environment, Health and Societies, Brunel University London, Uxbridge, Middlesex, UB8 3PH, United Kingdom; b AstraZeneca, Brixham Environmental Laboratory, Freshwater Quarry, Brixham, Devon, TQ5 8BA, United Kingdom; c Institute of Environment, Health and Societies, Brunel University London, Uxbridge, Middlesex, UB8 3PH, United Kingdom

**Keywords:** Ibuprofen, Biological read-across, Blood plasma, Prostaglandin E metabolite, Gene expression

## Abstract

Human pharmaceuticals present in the environment have the potential to cause adverse effects on non-target organisms. The “read-across hypothesis” stipulates that pharmaceuticals will exhibit similar biological effects across species (e.g. human and fish) if the molecular target has been conserved and the effective drug concentrations are reached (C_max_). We tested this hypothesis by evaluating if ibuprofen, a non-selective inhibitor of prostaglandins and the cyclooxygenase (COX) enzyme, can mimic its primary effect in humans, on fish, at comparable plasma concentrations. The endpoints, prostaglandin E metabolite (PGEM) levels and the mRNA expression of COX (*ptgs)* gene, were measured in the gills of control and exposed fathead minnows (*Pimephales promelas*), using enzyme-immunoassay and quantitative real-time PCR (qPCR). Fish were exposed, for 24–72 h, to measured water concentrations of 9 (n = 12), 370 (n = 40) and 470 μg ibuprofen/L (n = 12). Water and blood plasma concentrations were determined using LC-MS/MS. Results showed that PGEM levels in fish exposed to 370 and 470 μg ibuprofen/L were significantly decreased compared to control fish, when mean plasma ibuprofen concentrations were 1.8–5.6-fold below the C_max_. The plasma ibuprofen concentrations and PGEM levels varied greatly between individuals. In fish exposed to 9 μg ibuprofen/L, when the mean plasma ibuprofen concentration was 224-fold below C_max_, no change in PGEM levels was observed. These data provide evidence for the read-across hypothesis, but suggest establishing a direct dose-response between internal plasma and PGEM is difficult, and would require significantly larger numbers of fish to overcome the inter-individual variation.

## Highlights

• Study provides evidence for the “read-across hypothesis”.
• Ibuprofen decreased “prostaglandin E metabolite” (PGEM) levels in fathead minnows.
• “Mode-of-action” response seen in fish at plasma concentrations similar to the C_max_.
• Large inter-individual variation in plasma ibuprofen and PGEM levels observed.

## 1 Introduction

The presence of pharmaceuticals in the aquatic environment, and their real and potential effects on living organisms, have become major research topics. One important issue to resolve is how best to prioritize pharmaceuticals, so that research is focused on those that present the greatest risk to living organisms. Various strategies have been proposed (Roos et al., 2012). One strategy is to base the ranking of pharmaceuticals on how close the plasma concentrations of wildlife, such as fish, are to the effective drug concentrations in humans, provided that the molecular drug target (i.e. receptor or enzyme) has been conserved: this is the basis of the read-across hypothesis (Huggett et al., 2003, Rand-Weaver et al., 2013, Tanoue et al., 2015). Currently, little information is available to support the use of the read-across hypothesis to predict the environmental (e.g. river) concentrations of pharmaceuticals that will cause effects to fish. A recent publication (Margiotta-Casaluci et al., 2014) has shown that the anti-depressant fluoxetine affects behavior of fish only when their plasma concentration is within the human therapeutic range. However, the range of fluoxetine concentrations reported to affect various species of aquatic organisms is very wide (Sumpter et al., 2014), suggesting that the read-across hypothesis may not be universally applicable. To assess the robustness of the read-across hypothesis, we investigated the uptake of the human pharmaceutical ibuprofen by fish and the internal (blood plasma) concentrations required to elicit a mode-of-action related effect.

Ibuprofen is a non-selective inhibitor of the cyclooxygenase (COX) enzyme. There are two mammalian isoforms, COX 1 and COX 2, which are encoded by the *ptgs 1* and *ptgs 2* genes, respectively. During inflammation, the COX 2 enzyme is rapidly induced, increasing the biosynthesis of prostanoids, primarily prostaglandin E_2_ (PGE_2_). PGE_2_ has been identified in teleost fish species (e.g. zebrafish [*Danio rerio*] (Grosser et al., 2002, Morthorst et al., 2013); rainbow trout [*Oncorhynchus mykiss*] (Knight et al., 1995) and bluntnose minnow [*Pimephales notatus*] (Bhandari and Venables, 2011)), suggesting that the human drug target is conserved in fish.

Ibuprofen is a widely prescribed non-steroidal anti-inflammatory drug (NSAID), frequently detected in both wastewater effluents and rivers (Hughes et al., 2013, Osorio et al., 2015), typically in the low μg/L to ng/L range. There is an increasing body of evidence showing the potential for NSAIDs to perturb physiological processes in aquatic vertebrates, such as cortisol synthesis and osmoregulation (Gravel et al., 2009, Gravel and Vijayan, 2007), hematological changes (Saravanan et al., 2012) and fish reproduction and development (David and Pancharatna, 2009, Flippin et al., 2007). Recent studies, investigating possible effects of ibuprofen on fish, have suggested that adverse effects on the reproductive axis occur at concentrations of less than or equal to 0.1 μg/L (Ji et al., 2013) which, if repeatable, would indicate that ibuprofen poses a significant threat to populations of wild fish.

We tested the read-across hypothesis, by determining if ibuprofen inhibits PGE_2_ synthesis in fish, mimicking its primary effect in humans, and if so, at what water concentration. Water and plasma concentrations of ibuprofen were measured, as well as gill prostaglandin metabolite levels. By doing so, we assessed the validity of the read-across hypothesis as a means of predicting the water concentration of a pharmaceutical required to induce the anticipated mode-of-action related effect. We also included a water concentration that was not expected to produce the effective plasma concentrations in fish, in order to fully validate the read-across hypothesis.

## 2 Materials and methods

### 2.1 Fish husbandry

Adult (4–6 months), all male, fathead minnows were bred and held at AstraZeneca's Brixham Environmental Laboratory (Devon, UK), under a flow-through system at a water temperature of 25 ± 1 °C and a 16:8 light/dark photoperiod. Fish were fed (before exposures) twice daily with food pellet (Biomar, Brande, Denmark) and frozen adult brine shrimp (*Artemia sp.*). Work was conducted under license granted by the UK Home Office under the Animals (Scientific Procedures) Act 1986.

### 2.2 Test chemicals

Ibuprofen (≥98% purity, CAS:15687-27-1) was purchased from Sigma-Aldrich (Dorset, UK) and stored at room temperature. Ibuprofen stock solutions were prepared in acetone (CAS No. 67-64-1; ≥99% purity; Fisher Scientific, Loughborough, UK). Dechlorinated mains water (dilution water) was used to dilute the ibuprofen stock solutions to nominal test concentrations (5, 100, 270, 350, 370 and 500 μg/L) in glass tanks with a working volume of 45 L. A solvent control (SC) was also included. The acetone concentration was maintained at 0.0016% and therefore did not exceed 0.01%, as recommended by OECD guidelines (OECD, 1992).

### 2.3 Ibuprofen experiments

A (two-part) range-finder was conducted to assess the uptake of ibuprofen into fish blood plasma and to establish the water concentration(s) required to produce human therapeutic plasma concentrations (C_max_) in fish. Fathead minnows (n = 96; (wet) weight: 2.89 ± 0.53 g; (standard) length: 52.6 ± 4.04 mm) were first exposed, over 3–96 h, to SC (n = 16) and nominal water concentrations of 100 and 500 μg ibuprofen/L (n = 16 per concentration), using continuous flow-through conditions, and sampled (n = 4) after 3, 24, 48 and 96 h. In the second part, fathead minnows were exposed, over 24–96 h, to SC (n = 16) and nominal water concentrations of 270 and 370 μg ibuprofen/L, using continuous flow-through conditions, and sampled (n = 4) after 24, 48, 72 and 96 h.

Following the results of the range-finder, a suitable water (exposure) concentration was selected (one which was expected to produce fish plasma concentrations close to the C_max_) in order to examine prostaglandin metabolite levels in fish. Using a larger sample size, fathead minnows (n = 50; weight: 2.94 ± 0.80 g; length: 53.9 ± 4.27 mm) were exposed to SC (n = 10) and the nominal water concentration of 350 μg ibuprofen/L (n = 40) for 72 h, using continuous flow-through conditions, and then sampled.

In a third (smaller) experiment, designed to test the hypothesis that a low concentration of ibuprofen (one that would produce a fish plasma concentration well below the C_max_) would not cause an effect on prostaglandin metabolite levels, fathead minnows (n = 45; weight = 2.74 ± 0.47 g; length = 50.9 ± 4.03 mm) were exposed for 24–72 h to SC (n = 9) and nominal water concentrations of 5 μg ibuprofen/L (n = 18) or 350 μg ibuprofen/L (n = 18), using static conditions. The 5 μg/L concentration was not expected to produce the effective plasma concentrations in fish and the 350 μg/L was included to test the repeatability of the results of the earlier experiment. Fish were sampled after 24, 48 and 72 h (SC, n = 3; 5 μg/L, n = 6; and 350 μg/L, n = 6 at each time point). A static renewal design was used because it is much easier, uses lesser amounts of test chemical (ibuprofen) and is more cost-effective than it is to run a flow-through experiment. However, under ideal conditions, a flow-through would have been used.

### 2.4 Exposure conditions and water sampling

The dilution water was maintained at 25 ± 1 °C, dissolved oxygen (DO) ≥80% air saturation and pH at 7.4 ± 1. For the continuous flow-through regime, dilution water flowed into glass mixing vessels at a rate of 250 mL/min, and also received the stock test solution (via a syringe) at a rate of 0.004 mL/min in order to achieve the desired concentration in the tank. Separate glass lines from each mixing vessel supplied to the tank produced eight tank volume changes per day, which subsequently went to waste. For the static experiment, ibuprofen stock solutions were administered directly into the tanks at the beginning of the exposure. Temperature, pH, DO, alkalinity, hardness and conductivity were measured twice during the exposures. Fish were subjected to a 16:8 h (light:dark) photoperiod, with a 20 min dawn/dusk transition period and were not fed during the exposures. The water concentration of ibuprofen was measured daily. Water samples (5 mL) were collected using a pipette from the center of each tank.

### 2.5 Fish blood plasma and gill sampling

After exposure, fish were anaesthetized with MS-222 (500 mg/L buffered with 1 M sodium bicarbonate to pH 7.4 and aerated) and humanely sacrificed according to UK Home Office procedures. Fish were immediately wet weighed (g) and the standard length measured (mm). Each fish was terminated by removal of the brain and the tail removed using a scalpel to collect blood into a heparinized micro-capillary tube (Fisher Scientific, Loughborough, UK). The tubes were sealed at one end and centrifuged (12,300 × *g*, 4 min, 20 °C) to separate the plasma, which was stored on ice prior to chemical analysis. The gill filaments were dissected, separated into two tubes, snap frozen in liquid nitrogen and stored at −80 °C.

### 2.6 Chemical analysis of water and fish blood plasma

Ibuprofen concentrations were determined by reversed-phase Liquid Chromatography coupled with tandem Mass Spectrometry (LC-MS/MS) (see Appendix A). Initial chromatographic separation was carried out on a Dionex Ultimate 3000 instrument using a Gemini^®^ NX C18 column (50 × 2.0 mm, 3 μm, Phenomenex, CA, USA) maintained at 50 °C. The mobile phase consisted of 0.1% ammonia in water (eluent A) and 0.1% ammonia in methanol (eluent B), delivered at a flow rate of 500 μL/min. Ibuprofen was detected using an Ion Trap mass spectrometer (LTQ, Thermo Scientific, UK) with heated electrospray ionization. Data were acquired and processed using Xcalibur™ software (Thermo Scientific, UK). An internal standard, ibuprofen-d_3_ (CAS No. 121662-14-4, ≥98% purity; Sigma-Aldrich, Dorset, UK) was used for the quantification of ibuprofen in plasma samples (spiked with 80 μg/L ibuprofen-d_3_). The samples were analyzed in duplicate.

### 2.7 Measurement of prostaglandin E metabolite (PGEM)

PGE_2_ metabolites in the gills were measured using the Prostaglandin E Metabolite EIA Kit (Cayman Chemical Company, Ann Arbor, MI, USA). This kit converts unstable PGE_2_ metabolites into a single, stable derivative (PGEM) that can be more easily quantified. Briefly, frozen gill tissues (∼20 mg) were lysed in 500 μL of homogenization buffer (0.1 M phosphate, pH 7.4, 1 mM EDTA containing 10 μM indomethacin to prevent *ex vivo* formation of prostaglandins) using a tissue lyser (Tissue Lyser II, QIAGEN, Manchester, UK) and centrifuged (8000 × g, 10 min, 4 °C). Up to 120 μL of the supernatant was removed for protein quantification. The remaining supernatant was incubated at −20 °C for 60 min with 4 × volumes of ice-cold acetone and centrifuged (400 × g, 5 min, room temperature) to pellet the protein. The supernatant was transferred to a clean tube and the acetone was evaporated under nitrogen. The samples were re-suspended in enzyme-immunoassay buffer (0.1 M phosphate solution, pH 7.4, 0.1% bovine serum albumin, 0.4 M NaCl, 1 mM EDTA and 0.01% sodium azide). The PGEM standard solution (supplied in the kit) and samples were derivatized overnight at 37 °C. The PGEM concentration in each sample (assayed in triplicate) was determined from an 8-point standard curve (standards assayed in duplicate; 0.39–50 pg/mL). The curve regression coefficient (r^2^) was 0.9559. The PGEM level was normalized against the sample protein concentration (mg/mL) determined using the QuantiPro™ bicinchoninic acid (BCA) assay (Sigma-Aldrich, Poole, UK), and is expressed as pg/mg protein. Wild-type mouse lung tissue obtained from Dr Pook's group, Brunel University London, was used as an additional positive control to ensure correct sample preparation.

### 2.8 Measurement of *cyclooxygenase* (*ptgs*) gene expression

Total RNA was isolated from gills (∼20 mg) using the GenElute™ Mammalian Total RNA Miniprep kit (Sigma-Aldrich, Dorset, UK), treated with DNase I (Sigma-Aldrich, Dorset, UK) and reverse transcribed to cDNA using the iScript™ cDNA Synthesis Kit (Bio-Rad Laboratories, Hertfordshire, UK). Nuclease-free water was added to the RNA template as a negative control. Quantitative real-time PCR (qPCR) was carried out using the ABI Prism^®^ 7900 HT real-time PCR instrument (Applied Biosystems, Life Technologies, Paisley, UK). The primers used to amplify *ptgs* genes were designed from template gene fragments that we previously isolated in the fathead minnow (Suppl. Table S1). Each 25 μL PCR reaction consisted of cDNA (50 ng/μL), 2× QuantiFast SYBR Green mastermix (QIAGEN, Manchester, UK), forward and reverse primers (0.4 μM) and nuclease-free water. The reactions were assayed in duplicate. Nuclease-free water was added to the no-template (negative) controls. Amplification was carried out using the following cycling conditions: initial denaturation at 95 °C for 5 min, followed by 40 cycles at 95 °C for 10 s and 60 °C for 30 s. Melting curve analysis was used to check the specificity of the primers, and the amplicons were cloned and sequenced to confirm their identities. The mRNA levels of the *ptgs* genes were normalized against *β-actin*. Relative mRNA expression was calculated using the comparative (2^−ΔΔCt^) method (Livak and Schmittgen, 2001). The amplification efficiency for each primer pair was determined by the slope of the Ct standard curve.

### 2.9 Measured vs. modeled fish plasma concentrations

Measured plasma ibuprofen concentrations, from fish exposed in the range-finder, were compared with plasma concentrations modeled using the Fish Plasma Model (FPM) (Huggett et al., 2003), based on the non-kinetic bioconcentration model proposed by Fitzsimmons et al. (2001). The fish plasma concentration was estimated using the Log *K*_ow_ (3.80) reported by Brown et al. (2007) (Eq. 1). Many pharmaceuticals are amenable to ionization at different pH's and therefore use of the Log D (0.45 at pH 7.4, predicted using ACD/Labs, Toronto, ON, Canada) was also evaluated (Eq. 2). The fish plasma concentration was calculated using the measured water concentration over 96 h (Eq. 3).

(1) $$\mathrm{Log}P_{\text{blood}:\text{water}}=0.73\times \mathrm{Log}K_{\text{octanol}:\text{water}}-0.88$$

(2) $$\mathrm{Log}P_{\text{blood}:\text{water}}=0.73\times \mathrm{Log}D_{7.4}-0.88$$

(3) $$\left[\text{Fish}\;\text{Plasma}\right]=\left[\text{Measured}\;\text{Water}\right]\times P_{\text{blood}:\text{water}}$$

### 2.10 Statistics

Results are presented as mean ± standard deviation (sd). Statistical analysis and graphs were created using GraphPad Prism 6 (GraphPad Software, Inc). Statistical significance was tested using unpaired *t-tests* or one-way ANOVA followed by a multiple comparison test (at *p* < 0.05, *p* < 0.001 or *p* < 0.0001). The relationship between plasma ibuprofen and PGEM level was examined using regression analysis.

## 3 Results

### 3.1 Measured water and blood plasma ibuprofen concentrations

In the range-finder, the mean measured water concentrations (over 96 h) were 105 ± 2, 278 ± 70, 409 ± 26 and 502 ± 56 μg ibuprofen/L, which were close to the nominal values (105, 103, 111 and 101%, respectively, of 100, 270, 370 and 500 μg ibuprofen/L) (Suppl. Table S2). Ibuprofen measurements in the water of SC tanks were below the Limit of Detection (LOD; ≤ 2.5 μg/L). Ibuprofen entered into fathead minnow blood plasma, but large inter-individual variations were observed (Suppl. Table S3). The LOD for ibuprofen measurements in the plasma of SC fish was ≤30 μg/L. The mean plasma ibuprofen concentrations (over 3–96 h) in fathead minnows exposed to nominal 100 and 500 μg/L were 657 ± 183 μg/L and 106,047 ± 71,291 μg/L, respectively. The mean plasma ibuprofen concentrations in fish exposed to nominal 270 and 370 μg/L (over 24–96 h) were 14,409 ± 22,084 μg/L and 40,540 ± 36,100 μg/L, respectively, closer to the human therapeutic plasma concentrations (C_max_; 15,000–30,000 μg/L) (Schulz et al., 2012).

Therefore, the nominal water concentration of 350 μg/L was selected as a suitable exposure concentration for obtaining the C_max_ in fish in the subsequent experiments.

In the second experiment, fish were exposed to the mean measured water concentration (over 24–72 h) of 370 (368 ± 4) μg ibuprofen/L (105% of nominal concentration of 350 μg/L) (Suppl. Table S2). This resulted in a mean plasma ibuprofen concentration of 8370 ± 5456 μg/L, which was 1.8-fold below the lowest C_max_ (15,000 μg/L).

In the third experiment, fish were exposed to mean measured water concentrations (over 24–72 h) of 9 (±1) μg ibuprofen/L and 470 (473 ± 9) μg ibuprofen/L (181% and 135% of their respective nominal concentrations of 5 and 350 μg ibuprofen/L). This was a static water exposure and the discrepancy between the nominal and measured water concentrations was due to a dilution error. These water concentrations resulted in mean plasma ibuprofen concentrations of 67 ± 22 μg ibuprofen/L and 2680 ± 1605 μg ibuprofen/L, and were 224-fold and 5.6-fold below the lowest C_max_, respectively.

It is unclear why exposure to 470 μg ibuprofen/L resulted in a lower (mean) plasma ibuprofen concentration than exposure to 370 μg ibuprofen/L (2680 vs. 8370 μg ibuprofen/L). However, even though the mean plasma ibuprofen concentrations were different in the two experiments, there was some overlap as a consequence of the high fish-to-fish variability and unequal sample sizes (n = 12 vs. n = 40). The difference in the plasma concentrations could also be related to the static exposure regime utilized in the third experiment, as opposed to the flow through regime used in the previous experiments. However, the water (exposure) concentrations measured in the static experiment were generally stable (but higher than expected, due to a dilution error, as previously stated) over the 72 h (467 μg/L, 484 μg/L and 469 μg/L at 24, 48 and 72 h, respectively). The weight of the fish in the static experiment was slightly lower (but not significantly) than in the range-finder and experiment 2. The biomass of fish (g/L) in the SC, 5 and 350 μg ibuprofen/L tanks was 0.47, 1.0 and 1.1 g/L, respectively.

Hereafter, concentrations are expressed as nominal for the range-finder, and measured for the second (370 μg ibuprofen/L) and third experiments (9 and 470 μg ibuprofen/L) (as measured concentrations were more than 20% above the nominal).

### 3.2 PGEM levels in fathead minnow gill tissues

**Fig. 1:**
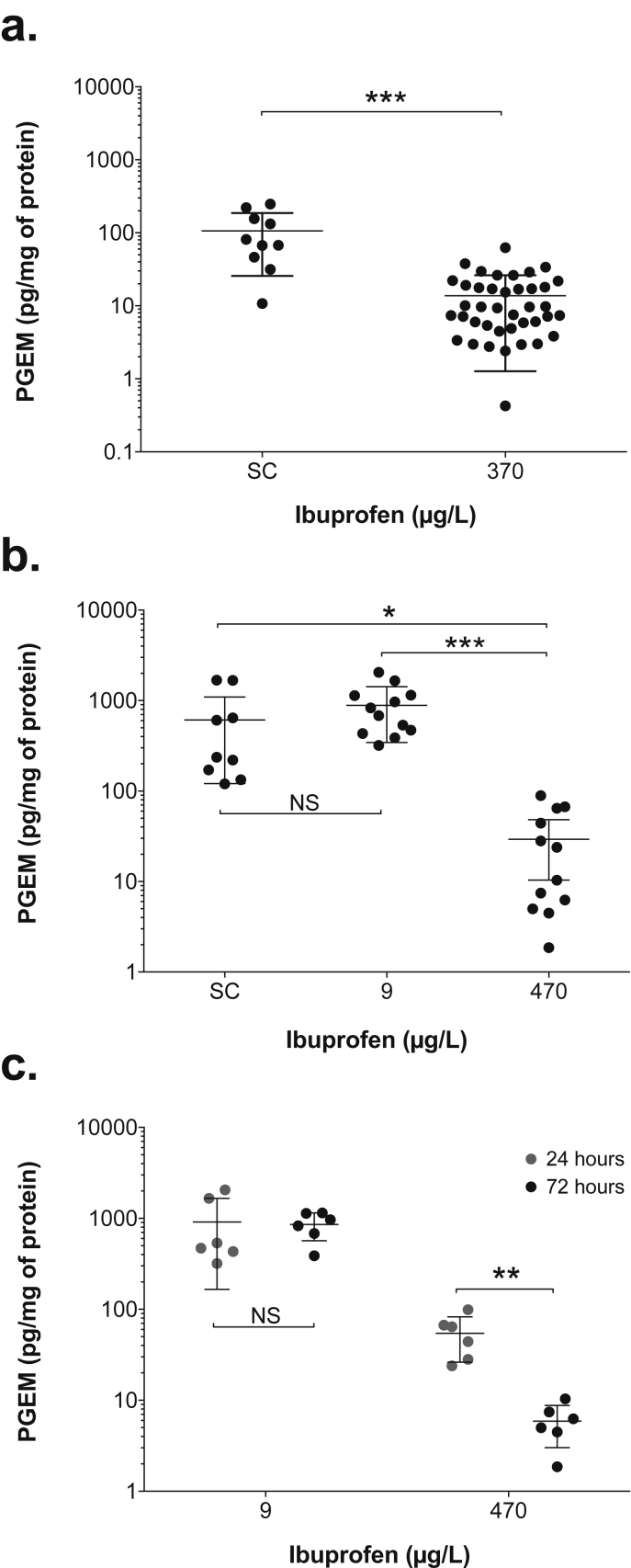
**Prostaglandin E metabolite (PGEM) levels in the gills of fathead minnows exposed to solvent (SC) and ibuprofen**. Fish were exposed to (a) SC (n = 10) and 370 μg/L (n = 40) for 72 h and (b) SC (n = 9), 9 μg/L (n = 12) and 470 μg/L (n = 12) for 24, 48 (SC fish only) and 72 h (data combined). In (c), data from 9 and 470 μg/L are shown for 24 or 72 h (n = 6 for each time point for each concentration). Each dot represents an individual fish within the treatment group, along with the mean ± sd. * indicates significance level of *p* < 0.05, ** of *p* < 0.001 and *** of *p* < 0.0001. (Results presented are from experiments 2 and 3; and are displayed as such so that the data from every fish can be readily visualized).

The mean PGEM level (14 ± 12.5 pg/mg) was significantly decreased (*p* = < 0.0001) in the gills of fathead minnows exposed, for 72 h, to 370 μg ibuprofen/L (experiment 2), when compared to the SC (106 ± 80 pg/mg) (Fig. 1a). To determine if the effects on PGEM could be replicated, and if an effect could be seen at a water concentration that was not expected to produce the effective fish plasma concentrations, fathead minnows were exposed to (measured) 9 μg ibuprofen/L (and 470 μg ibuprofen/L) over 24–72 h (experiment 3; data from exposed fish at 48 h not presented).

The mean PGEM level (884 ± 540 pg/mg) was not significantly different (*p* = 0.30) in the gills of fish exposed to 9 μg ibuprofen/L, when compared to the SC (609 ± 635 pg/mg) (Fig. 1b). The mean PGEM level (29 ± 30 pg/mg) was significantly decreased (*p* = 0.005) in the gills of fish exposed to 470 μg ibuprofen/L, when compared to the SC, and 9 μg ibuprofen/L (*p* < 0.0001) (Fig. 1b).

The PGEM levels were highly variable in the SCs (ranging between 10 and 248 pg/mg and 120–1680 pg/mg in the two experiments) and in the exposed groups (between 0.4- 62 and 1.9–89 pg/mg in the 370 and 470 μg ibuprofen/L groups, respectively).

Fig. 1c demonstrates that the PGEM level was significantly decreased (*p* = 0.001) between 24 and 72 h in fish exposed to 470 μg ibuprofen/L, indicating a time-dependent effect, similar to that which occurs in humans. There was no significant difference (*p* = 0.9) in PGEM level between 24 and 72 h in fish exposed to 9 μg ibuprofen/L.

### 3.3 The relationship between plasma ibuprofen and PGEM levels

**Fig. 2:**
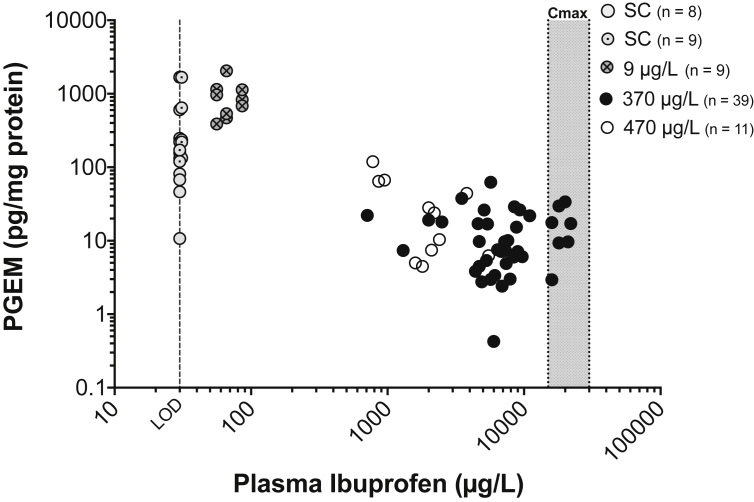
**The relationship between plasma ibuprofen (μg/L) and gill Prostaglandin E metabolite (PGEM) levels (pg/mg) in fathead minnows**. Fish were exposed for 72 hours to solvent (SC) and 9, 370 and 470 μg ibuprofen/L (n = number of fish plasma samples analyzed; each dot represents an individual fish). Ibuprofen measurements in the plasma of SC fish were below the LOD (<30 μg/L). C_max_ denotes the human therapeutic plasma concentrations (15,000–30,000 μg/L). The mean plasma ibuprofen level in fish exposed to 9 μg/L was 224-fold below the lowest C_max_ (15,000 μg/L). The mean plasma ibuprofen level in fish exposed to 370 and 470 μg/L was 1.8 and 5.6-fold below the lowest C_max_. (Results presented are from experiments 2 and 3).

A dose-response between plasma ibuprofen and PGEM levels was seen between fish exposed to 9 μg ibuprofen/L (no mode-of-action related response) and 370 and 470 μg ibuprofen/L (mode-of-action related response observed) (Fig. 2). However, there was no clear relationship between the plasma ibuprofen and the magnitude of the effect (i.e. amount of PGEM inhibition) in fish exposed to 370 and 470 μg ibuprofen/L (r^2^ = 0.01).

The PGEM levels in fish exposed to 9 μg ibuprofen/L were similar to the PGEM levels measured in the SC fish, when the mean plasma ibuprofen concentration (67 μg/L) was 224-fold below the lowest C_max_. Decreased PGEM levels were seen in fish exposed to 370 and 470 μg ibuprofen/L, when the mean plasma ibuprofen concentration was 1.8–5.6 fold below the lowest C_max_. The reduced PGEM levels were observed in fish with plasma ibuprofen concentrations spanning over 30-fold (from 710 to 22,000 μg ibuprofen/L). These results demonstrate that ibuprofen can inhibit PGEM levels over a range of plasma concentrations, including concentrations up to 21-fold below the lowest C_max_.

There was large inter-individual variation in plasma ibuprofen (30-fold, 710–22,000 μg/L) and PGEM levels (∼50-fold, 2.4–118 pg/mg). The PGEM levels were also highly variable in the SC fish (∼170-fold, 10–1680 pg/mg).

### 3.4 *Cyclooxygenase (ptgs)* gene expression in gill tissues

We identified three isoforms of *ptgs* genes in the fathead minnow, a situation similar to that in zebrafish (Ishikawa et al., 2007). In experiment 2, the mean expression of *ptgs 1* was unchanged in gills of exposed fish (compared to SC fish), whereas the *ptgs 2a* and *ptgs 2b* genes trended towards up-regulation, with *ptgs 2b* being close to significance (*p* values = 0.10, 0.12 and 0.07, respectively) (Suppl. Figure S1).

To establish if there was a dose-response between gene expression and drug plasma concentration, *ptgs* gene expression in gills and plasma ibuprofen in exposed fish was also examined; however, no correlation was observed (Suppl. Figure S2), nor between *ptgs* gene expression and PGEM levels in gills (Suppl. Figure S3).

### 3.5 Accuracy of the Fish Plasma Model (FPM)

**Fig. 3:**
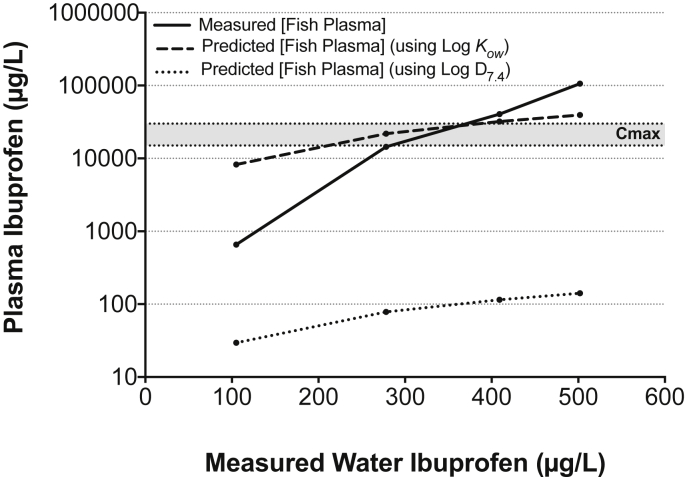
**Comparison of measured and predicted ibuprofen concentrations in fathead minnow plasma**. Fish were exposed to mean measured ± sd water concentrations of 105 ± 2 μg/L (over 3–96 hours), 278 ± 70 μg/L (24–96 hours), 409 ± 26 μg/L (24–96 hours) and 502 ± 56 μg/L (3–96 hours). The plasma ibuprofen concentration was calculated using both Log *K*ow and Log D_7.4_. The modeled values were based on the equation described by Fitzsimmons et al. (2001) and the FPM proposed by Huggett et al. (2003). C_max_ denotes the human therapeutic plasma concentrations (15,000–30,000 μg/L). (Results presented are from the range-finder).

The FPM, modeled using Log *K*_ow_, over-estimated the plasma ibuprofen concentration by 12-fold at 100 μg ibuprofen/L and under-estimated the plasma ibuprofen concentration by 3-fold at 500 μg ibuprofen/L (Fig. 3). The FPM most accurately estimated the plasma ibuprofen concentration at 270 and 370 μg ibuprofen/L, the concentrations at which the C_max_ was reached. The FPM severely under-estimated the plasma ibuprofen concentration in fish at all tested water concentrations when the Log D_7.4_ was used.

## 4 Discussion

The aim of this study was to assess the validity of the read-across hypothesis, by examining the effects of ibuprofen on fish. We addressed the criteria of the read-across hypothesis, as outlined by Rand-Weaver et al. (2013), and confirmed the water concentration(s) and blood plasma concentration(s) of ibuprofen, linked the exposure to a specific mode-of-action biological effect expected at the C_max_ (by measuring PGEM, surrogate marker for PGE_2_), and demonstrated that this effect was seen at plasma concentrations similar to (1.8- to 5.6-fold below) the lowest C_max_ (15,000 μg/L). In humans, the therapeutic effects of ibuprofen (i.e. perceptible pain relief) have been reported at plasma ibuprofen levels of 6800–10,100 μg/L (20 min post 400 mg dosing), and maximal pain relief was confirmed at 30 min, when plasma levels were 14,800–18,900 μg/L (Mehlisch and Sykes, 2013). These findings suggest that the on-set of therapeutic effects can occur at blood plasma concentrations below the stated C_max_ (15,000–30,000 μg/L) (Schulz et al., 2012), and may explain why the anticipated mode-of-effect was observed in fish before the C_max_ (peak plasma concentration) was reached.

A mode-of-action related effect on PGEM was observed in ibuprofen-exposed fish when the mean blood plasma concentration was 1.8- to 5.6-fold, but not 224-fold, below the lowest C_max_ (15,000 μg/L). These findings provide evidence in support of the read-across hypothesis and demonstrate that pharmaceuticals can exert similar target-mediated pharmacological effects in fish as they do in humans, at similar blood plasma concentrations. Our findings are consistent with the mode-of-action relevant behavioral effects of sertraline and fluoxetine reported in fish at plasma concentrations similar to human therapeutic concentrations (Margiotta-Casaluci et al., 2014, Valenti et al., 2012) and the effects of the synthetic progestagen, levonorgestrel (Runnalls et al., 2015). Collectively, these studies provide experimental evidence for the read-across hypothesis, identified as lacking by Rand-Weaver et al. (2013). In fish exposed to the lowest tested concentration (9 μg ibuprofen/L), when the mean plasma ibuprofen concentration was 224-fold below the lowest C_max_, no mode-of-action effect on PGEM levels was seen.

The mode-of-action of ibuprofen is related to its non-specific inhibition of prostanoids, via the inhibition of the COX enzymes. Consistent with our findings, the effects of ibuprofen on PGE_2_ levels have also been demonstrated in zebrafish (whole body homogenates or ovaries), following 7-d exposure to 21–506 μg/L (Morthorst et al., 2013) and in bluntnose minnows, following exposure to 50 μg/L and 100 μg/L ibuprofen, which resulted in a significant reduction in gill PGE_2_ concentrations (Bhandari and Venables, 2011). In the latter study, the lower (5 and 25 μg ibuprofen/L) concentrations tested did not result in a significant reduction in PGE_2_ compared to the controls, similar to our findings. The effect of ibuprofen on PGEM reduction in fish exposed to 470 μg ibuprofen/L was also more pronounced at 72 than 24 h. Such time-dependency has not previously been reported in fish, but is in agreement with data from humans (Giagoudakis and Markantonis, 2005, Rane et al., 1978, Rome and Lands, 1975).

Exposure to stressors can lead to pain and inflammation, resulting in a proliferated increase in prostanoids, primarily PGE_2_ (Vane, 1971). PGE_2,_ is chemically unstable and is rapidly converted *in vivo* to several PGE metabolites (13,14-dihydro-15-keto PGE_2_ and 13,14-dihydro-15-keto PGA_2_) (Ferreira and Vane, 1967). For this reason, blood and other animal or human samples often contain very little intact PGE_2_. Therefore, in this study, the measurement of PGE metabolites (PGEM) was used to provide an estimate of the actual (parent) PGE_2_ production. We investigated only the primary mammalian mode-of-action of ibuprofen, namely its ability to inhibit PGE_2_ synthesis, and showed that ibuprofen acts via this mode-of-action in fish also. The biological functions of PGE_2_ in fish are not well characterized, and the consequences of prostanoid inhibition in fish are presently unknown. However, prostanoids have been implicated in several “homeostatic” functions in fish, including reproduction (Fujimori et al., 2011, Gonçalves et al., 2014, Lister and Van Der Kraak, 2008, Sorbera et al., 2001) glucose metabolism (Busby, 2002) and immunity (Gómez-Abellán and Sepulcre, 2016). Further work is necessary to investigate how NSAIDs affect fish physiology and until those consequences are known, it is not possible to develop a meaningful Adverse Outcomes Pathway for this group of pharmaceuticals.

In this study, we examined the effect of ibuprofen on the PGEM level in the gills, which we considered to be a suitable tissue for examining mode-of-action effects. In our preliminary work (data not shown) we also measured the PGEM level in liver and brain (the plasma was not used as it was required for chemical analysis). However, due to the limited amount of starting material and lower amounts of PGE_2_, the tissues had to be pooled (n = 1), making any effect very difficult to ascertain. The PGEM level was easier to measure in gill tissue, in agreement with the results of others (Bhandari and Venables, 2011, Knight et al., 1995) who have reported a higher abundance, and the most consistent measurable levels of PGE_2_, in this tissue. However, as prostaglandin levels in one tissue (gills) do not represent global levels in fish, further studies should examine PGEM levels in blood plasma and other tissues.

The PGEM levels were highly variably in solvent control and ibuprofen-exposed fish. The variation in control groups suggests that factors other than drug exposure can also modulate PGEM production in “healthy” fish tissues. It is difficult to pinpoint the exact cause of such variation, since the controllable factors, including sex (all male) and age (4–6 month), were the same, and the same handling and sampling procedures were used throughout. It has been suggested that changes in dietary fatty acids can result in extensive alteration in the profile of prostanoids in fish tissues (Norambuena et al., 2012). However, in this study, the fish were not fed. We speculate that the variation observed in the control fish may be normal and represents the natural variation in a population of laboratory-bred fathead minnows. Further work is needed to gain an understanding of the “expected level” of the biological variation within a sample population. In order to reduce the uncertainty in the data, the sample size (n) could be increased, although it was reasonably high in the studies reported here.

There was a dose-response between plasma ibuprofen and PGEM levels between fish exposed 9 μg/L and 370 or 470 μg/L. However, as a consequence of testing only a limited number of concentrations of ibuprofen (9, 370 and 470 μg/L), which were not evenly distributed, it is not possible to know exactly where the concentration-response relationship lies and what shape it would take, and thus what type of regression analysis would best describe that relationship. Given the large inter-individual variation in plasma ibuprofen (30-fold, 710–22,000 μg/L) and PGEM levels (50-fold, 2.4–118 pg/mg), there was only a 10-fold difference between the lowest measured plasma concentration (710 μg/L) in the 370 μg/L exposure group, and the mean plasma level (67 μg/L) in the 9 μg/L group. This indicates that there is a relatively narrow margin between the plasma concentrations at which no PGEM inhibition was observed, and at which PGEM was significantly decreased. Further work is needed to ascertain the lowest (water) exposure concentration at which effects on PGEM would be seen (i.e. between 9 and 370 μg/L).

The large inter-individual variation in plasma ibuprofen concentrations has a major impact on attempts to relate effective drug plasma concentrations with target-mediated effects in fish, and can be identified as a limitation of the read-across approach. Further work is needed to identify the expected level of variation in drug plasma concentrations, particularly in fish exposed to the same concentration of a drug. Certainly, in humans, variability in drug responses between individuals is well recognized (Wood, 2001), and therefore drugs exert their therapeutic effects over a range of concentrations (before adverse drug reactions occur) (Brune et al., 2010). This variability is largely influenced by drug pharmacokinetics and pharmacodynamics (Derendorf et al., 2000, Reigner et al., 1997, Sheiner and Steimer, 2000). For example, variations in plasma protein binding can affect the distribution of bound and free drug fractions in the body (Lin et al., 1987) and drug metabolism can be influenced by the presence of genetic polymorphisms (García-Martín et al., 2004). Much less is known about pharmacokinetics and pharmacodynamics in fish; for instance, the drug plasma-protein binding kinetics (Owen et al., 2007) are nearly always unknown. It is possible that differential plasma-protein binding and/or drug distribution into other tissues may account for why reduced (gill) PGEM levels were seen in fish with plasma ibuprofen concentrations ranging over 30-fold. However, this is only speculative. Another possible explanation is saturation of the active sites of the COX protein following NSAID treatment, which has been observed in rat models (Satterwhite and Boudinot, 1991). Ibuprofen is a weak, competitive non-selective inhibitor of the COX enzymes (Gierse et al., 1999), and therefore after 72 h of continuous exposure, an increase in the plasma ibuprofen concentration beyond a certain level (i.e. when all available active sites have been occupied) possibly would have no further effect on PGEM inhibition.

It is possible to use the relationship between plasma concentrations in humans (the C_max_), and in fish, in order to predict the likelihood for similar pharmacological responses (Huggett et al., 2003, Rand-Weaver et al., 2013, Schreiber et al., 2011). The plasma concentrations modeled using FPM (using the Log *K*_ow_) accurately mirrored the measured plasma concentration in fish exposed to 270 and 370 μg ibuprofen/L, and correctly predicted that the C_max_ would be reached at these water concentrations. These findings are in agreement with other studies (Fick et al., 2010, Margiotta-Casaluci et al., 2014, Nallani et al., 2016, Valenti et al., 2012) that have used the FPM to accurately (within an order of magnitude) predict the plasma concentrations of human pharmaceuticals in fish. In contrast to our findings, a recent study found Log D_7.0_ to be a better predictor of plasma bioconcentration in fish (Nallani et al., 2016) However, in that study, ibuprofen only weakly bioconcentrated (by 1.4-fold) into channel catfish plasma after 7-d exposure to measured water concentrations (314 ± 55 μg/L) (Nallani et al., 2011), whereas we show higher concentrations of ibuprofen accumulated in the plasma of fathead minnows. The FPM over (by 12-fold)- and under (by 3-fold)-estimated the plasma concentrations in fish exposed to 100 and 500 μg/L ibuprofen, respectively. It is not known why this was the case, however, potential sources of inaccuracy may arise from factors such as drug ionization, transporters, metabolism, excretion and/or plasma protein binding kinetics on drug uptake/plasma concentration. For example, the presence of steroid hormone binding globulins in teleost fish gills (Miguel-Queralt and Hammond, 2008) may increase the uptake rate of steroid pharmaceuticals above that predicted by the FPM. Despite the growing support for the use of the FPM, further experimental studies are still required in order to evaluate how well the concentration of a pharmaceutical in fish blood plasma can be theoretically modeled for a given water concentration.

The mammalian COX enzymes are encoded by the *ptgs 1* and *ptgs 2* genes. The additional *ptgs 2* (*2a* and *2b*) gene identified in the fathead minnow is likely a result of genome duplication in teleost fish (Havird et al., 2008). The expression levels of all *ptgs* genes were not significantly different between control and exposed fish (Suppl. Figure S1), although there was a trend towards up-regulation of the *ptgs* 2 genes (p = 0.07 for *ptgs* 2b). This result is in agreement with those of other studies reporting little or no transcriptional change in *ptgs* gene expression in zebrafish exposed to ibuprofen (Ji et al., 2013, Morthorst et al., 2013). In contrast, rainbow trout exposed for two weeks to 1.6–81.5 μg diclofenac/L (1.5–88% of the C_max_) had global hepatic gene expression changes consistent with the mode-of-action of NSAIDs (Cuklev et al., 2011). At plasma concentrations close to the C_max_, a number of genes functionally associated with inflammation and the immune response were differentially regulated in the liver, and the hepatic expression of the *ptgs 1* and *ptgs 2* genes was down-regulated. However, the lack of a concentration-related response raises questions about these results (Harris et al., 2014). In our study, the gene expression levels were highly variable between individual fish, and no relationship between gill *ptgs* expression and plasma ibuprofen was observed, suggesting that this is not a suitable biomarker for ibuprofen exposure. Given the lack of knowledge of what influences gene expression and the sensitivity of this as an end-point, more research is required before robust conclusions can be reached regarding the relationship between the expression of the *ptgs* genes and their product, the COX enzyme.

Ibuprofen has been reported in UK surface waters and final effluents at typical mean concentrations of 74 ng/L (Kasprzyk-Hordern et al., 2009) and 2.48 μg/L (Gardner et al., 2012), respectively. In this study, the 370 and 470 μg ibuprofen/L water concentrations produced plasma ibuprofen concentrations in fish closest to the C_max_ and induced the anticipated mode-of-action related effect. Thus, this effect occurred at a water concentration between 190 and 6300-fold higher than the environmental concentration. However, in reality, pharmaceuticals exist as complex mixtures (Sumpter, 2009, Vasquez et al., 2014), and there may be several prostanoid inhibitors present in the ng to μg/L range. Therefore, the potential for additive effects exist.

## 5 Conclusions

We demonstrate a mode-of-action related response on PGEM levels in fish exposed to ibuprofen, at blood plasma concentrations similar to those effective in humans. This study adds to an increasing body of research supporting the read-across hypothesis and FPM. However, we also highlight the importance of using large sample sizes, as large inter-individual variation was observed.
