# Supplementary material for: Testing the “read-across hypothesis” by investigating the effects of ibuprofen on fish
Source: Chemosphere. 2016 Nov;163:592–600. doi: 10.1016/j.chemosphere.2016.08.041 (PMC5034852; doi:10.1016/j.chemosphere.2016.08.041)
Supplement: Supplementary file 1 [file mmc1.docx]

**Supplementary Information & Appendix**

**For**

**Testing the “Read-across hypothesis” by investigating the effects of ibuprofen on fish.**

Alpa Patel,^a^  Grace H. Panter,^b,I^ Henry T. Trollope,^b,II^  Yohanna C Glennon,^b^  Stewart F. Owen,^b,III^  John P. Sumpter,^c,^* and Mariann Rand-Weaver.^a^

^a^ Biosciences, Institute of Environment, Health and Societies, Brunel University London, Uxbridge, Middlesex, United Kingdom, UB8 3PH.

^b^ Previous affiliation: AstraZeneca, Brixham Environmental Laboratory, Freshwater Quarry, Brixham, Devon, United Kingdom, TQ5 8BA.

^c^ Institute of Environment, Health and Societies, Brunel University London, Uxbridge, Middlesex, United Kingdom, UB8 3PH.

*Corresponding author: Prof John P. Sumpter. Institute of Environment, Health and Societies, Brunel University London, Uxbridge, Middlesex, United Kingdom, UB8 3PH. Email: [John.Sumpter@Brunel.ac.uk](mailto:John.Sumpter@Brunel.ac.uk). Tel: +44 (0)1895 266303.

**Table S1** Primers used to isolate *cyclooxygenase* (*ptgs)* genes in the fathead minnow.

**Table S2** details the nominal and measured water ibuprofen concentrations (μg/L) in the exposure tanks.

**Table S3** shows the measured plasma ibuprofen (μg/L) and blood plasma bioconcentration factor (BCF) in fathead minnows exposed, over 3-96 h, to nominal water concentrations of 100, 270, 370 and 500 μg ibuprofen/L (range-finder study).

**Figure S1** The (mean ± sd) cyclooxygenase (*ptgs*) gene expression in fathead minnows exposed for 72 h to 370 μg ibuprofen/L.

**Figure S2** explores the relationship between *ptgs* gene expression (in gills) and plasma ibuprofen concentration in fathead minnows exposed for 72 h to 370 μg ibuprofen/L.

**Figure S3** explores the relationship between *ptgs* gene expression and Prostaglandin E metabolite (PGEM) levels in the gills of fathead minnows exposed for 72 h to 370 μg ibuprofen/L.

**Appendix A** provides further information on the quantification of ibuprofen in water and fathead minnow blood plasma.

“**Table S1**. Primers used to isolate *cyclooxygenase* (*ptgs)* genes in the fathead minnow.”

| **Gene** | **Primer ID** | **Primer Sequence 5’- 3’** |
| --- | --- | --- |
| *ptgs 1* | External  *ptgs* *1* Forward | ACCTGGGRBGATGAGCAGCT |
|  | External  *ptgs* *1* Reverse | CCAAATGTGCTGGGCTTCCA |
|  | Internal  *ptgs* *1* Forward | CACTGGCACCCHCTBATGCC |
|  | Internal  *ptgs* *1* Reverse | AATGTGCTGGGCTTCCAGTA |
| *ptgs 2* | External  *ptgs* *2* Forward | ACTTCACCCACCAGTTCTTC |
|  | External  *ptgs* *2* Reverse | ATCAGTGGGTGCCAGTGGTA |
|  | Internal  *ptgs* *2* Forward | TCTGGCTGAGAGAACACAAC |
|  | Internal  *ptgs* *2* Reverse | ATGCGGTTCTGATACTGGAA |

There were no mRNA sequences available for the *ptgs* genes in the fathead minnow. Therefore, published data on *ptgs* gene sequences in zebrafish (*Danio rerio*) (Grosser et al., 2002; Ishikawa et al., 2007) and rainbow trout *(Oncorhynchus mykiss*) (Ishikawa et al., 2007) were used to search for homologues in other fish. The “Basic Local Alignment Search Tool” (BLAST) (http://blast.ncbi.nlm.nih.gov/Blast.cgi) retrieved both full length and partial mRNA sequences for *ptgs* isoforms (*ptgs 1, ptgs 2a* and *ptgs 2b*) which were aligned using a multiple sequence alignment tool (<http://www.ebi.ac.uk/Tools/msa/clustalo/>), to examine regions of highly conserved nucleotide bases. Two pairs of generic primers, one “external” and one “internal” set, were designed against highly conserved regions in order to increase the likelihood of isolating one *ptgs 1* and *ptgs 2* isoform in the fathead minnow. Both of the *ptgs 1* primer sets contained a degenerate (forward) primer. Degenerate primers contain base positions (underlined) that can be substituted for other known nucleotide bases. The “R” positions can be substituted with A or G, “B” with G or T or C and “H” with A or T or C bases. To confirm the specificity of the primers, the PCR products were cloned and sequenced to confirm their identities, and were analyzed for similarity to other *ptgs* gene sequences using BLAST. The primers were designed to amplify products of 100-200 bp in length, with a 40-60% GC content and melting temperature (Tm) of 60°C and were synthesized by Sigma Genosys Ltd (Suffolk, UK).

References:

Grosser, T., Yusuff, S., Cheskis, E., Pack, M.A., FitzGerald, G.A., 2002. Developmental expression of functional cyclooxygenases in zebrafish. Proc. Natl. Acad. Sci. 99, 8418–8423. doi:10.1073/pnas.112217799

Ishikawa, T., Herschman, H.R., 2007. Two inducible, functional cyclooxygenase-2 genes are present in the rainbow trout genome. J. Cell. Biochem. 102, 1486–1492. doi:10.1002/jcb.21368

Ishikawa, T.-O., Griffin, K.J.P., Banerjee, U., Herschman, H.R., 2007. The zebrafish genome contains two inducible, functional cyclooxygenase-2 genes. Biochem. Biophys. Res. Commun. 352, 181–187. doi:10.1016/j.bbrc.2006.11.007.

“**Table S2**. Nominal and measured (mean ± sd) water ibuprofen concentrations (μg/L) in exposure tanks (results presented are from range-finder, experiments 2 and 3). The measured water concentration (column 3) is the mean value of two samples.”

| **Nominal Water µg/L** | **Exposure h** | **Measured water µg/L** | **% of Nominal** |
| --- | --- | --- | --- |
| **Range-finder (flow-through)** | | | |
| 100 | 3 | 107 | 107 |
|  | 24 | 107 | 107 |
|  | 48 | 105 | 105 |
|  | 96 | 102 | 102 |
|  | **Mean (± sd)** | **105 (± 2)** | **105** |
| 270 | 24 | 285 | 105 |
|  | 48 | 179 | 66 |
|  | 72 | 343 | 127 |
|  | 96 | 307 | 114 |
|  | **Mean (± sd)** | **278 (± 70)** | **103** |
| 370 | 24 | 422 | 114 |
|  | 48 | 416 | 113 |
|  | 72 | 428 | 116 |
|  | 96 | 371 | 100 |
|  | **Mean (± sd)** | **409 (± 26)** | **111** |
| 500 | 3 | 539 | 108 |
|  | 24 | 419 | 84 |
|  | 72 | 518 | 104 |
|  | 96 | 532 | 106 |
|  | **Mean (± sd)** | **502 (± 56)** | **101** |
| **Experiment 2 (flow-through)** | | | |
| 350 | 24 | 363 | 104 |
|  | 48 | 370 | 106 |
|  | 72 | 370 | 106 |
|  | **Mean (± sd)** | **368 (± 4)** | **105** |
| **Experiment 3 (static)** | | | |
| 5 | 24 | 10 | 200 |
|  | 48 | 9 | 180 |
|  | 72 | 8 | 165 |
|  | **Mean (± sd)** | **9 (± 1)** | **181** |
| 350 | 24 | 467 | 134 |
|  | 48 | 484 | 138 |
|  | 72 | 469 | 133 |
|  | **Mean (± sd)** | **473 (± 9)** | **135** |

| **Exposure h** | **Measured water µg/L** | **Mean measured plasma**  **(*n,* ±sd) µg/L** | **Blood plasma BCF** |
| --- | --- | --- | --- |
| 3 | 107 | 897 (*n*=1) | 8 |
| 24 | 107 | 550 (*n*=1) | 5 |
| 48 | 105 | 697 (*n*=1) | 7 |
| 96 | 102 | 485 (*n*=1) | 5 |
| **Mean (± sd)** | **105 (± 2)** | **657 (± 183)** |  |
| 24 | 285 | 2000 (*n*=3, ± 1732) | 7 |
| 48 | 179 | 2620 (*n*=3, ± 1170) | 15 |
| 72 | 343 | 4429 (*n*=4, ± 2555) | 13 |
| 96 | 307 | 48,590 (*n*=4, ± 82,880) | 158 |
| **Mean (± sd)** | **278 (± 70)** | **14,409 (± 22,810)** |  |
| 24 | 422 | 2944 (*n*=4, ± 1386) | 7 |
| 48 | 416 | 2942 (*n*=4, ± 1594) | 7 |
| 72 | 428 | 139,104 (*n*=4, ± 121,065) | 325 |
| 96 | 371 | 17,170 (*n*=4, ± 20,362) | 46 |
| **Mean (± sd)** | **409 (± 26)** | **40,540 (± 66,050)** |  |
| 3 | 539 | 59,935 (*n*=1) | 111 |
| 24 | 419 | 31,800 (*n*=1) | 76 |
| 72 | 518 | 152,714 (*n*=1) | 295 |
| 96 | 532 | 179,739 (*n*=4, ± 126,138) | 338 |
| **Mean (± sd)** | **502 (± 56)** | **106,047 (± 71,291)** |  |

“**Table S3**. Measured plasma ibuprofen concentrations (μg/L) and blood plasma bioconcentration factor (BCF) in fathead minnows exposed, over 3-96 h, to nominal water concentrations of 100, 270, 370 and 500 µg ibuprofen/L (results presented from range-finder study only).”

The blood plasma samples collected from fathead minnows exposed to nominal 100 µg ibuprofen/L (3-96 h) and nominal 500 µg ibuprofen/L (3-72 h) were pooled (n= 1) due to limited volumes of plasma, as some was used for analytical method development. Individual plasma samples were collected from fathead minnows (n= 3-4) exposed to 270 and 370 µg ibuprofen/L (24-96 h) and 500 µg ibuprofen/L (at 96 h only). Brackets denote the number of fish plasma samples at each time point. The blood plasma bioconcentration factor (BCF) of ibuprofen from the water into blood plasma (column 4) was calculated using the mean measured plasma concentration divided by the measured water concentration.

“**Figure S1**. The (mean ± sd) cyclooxygenase (*ptgs*) gene expression in fathead minnows exposed for 72 h to 370 μg ibuprofen/L. The *ptgs* 1, *ptgs* 2a and *ptgs* 2b expression levels were measured in the gills of solvent control (SC, n= 10) and ibuprofen-exposed (n= 36-40) fish and were normalized to *β-actin*. The data from the exposed fish are expressed as fold change relative to the (average) gene expression level in the SC fish (represented as 1).”


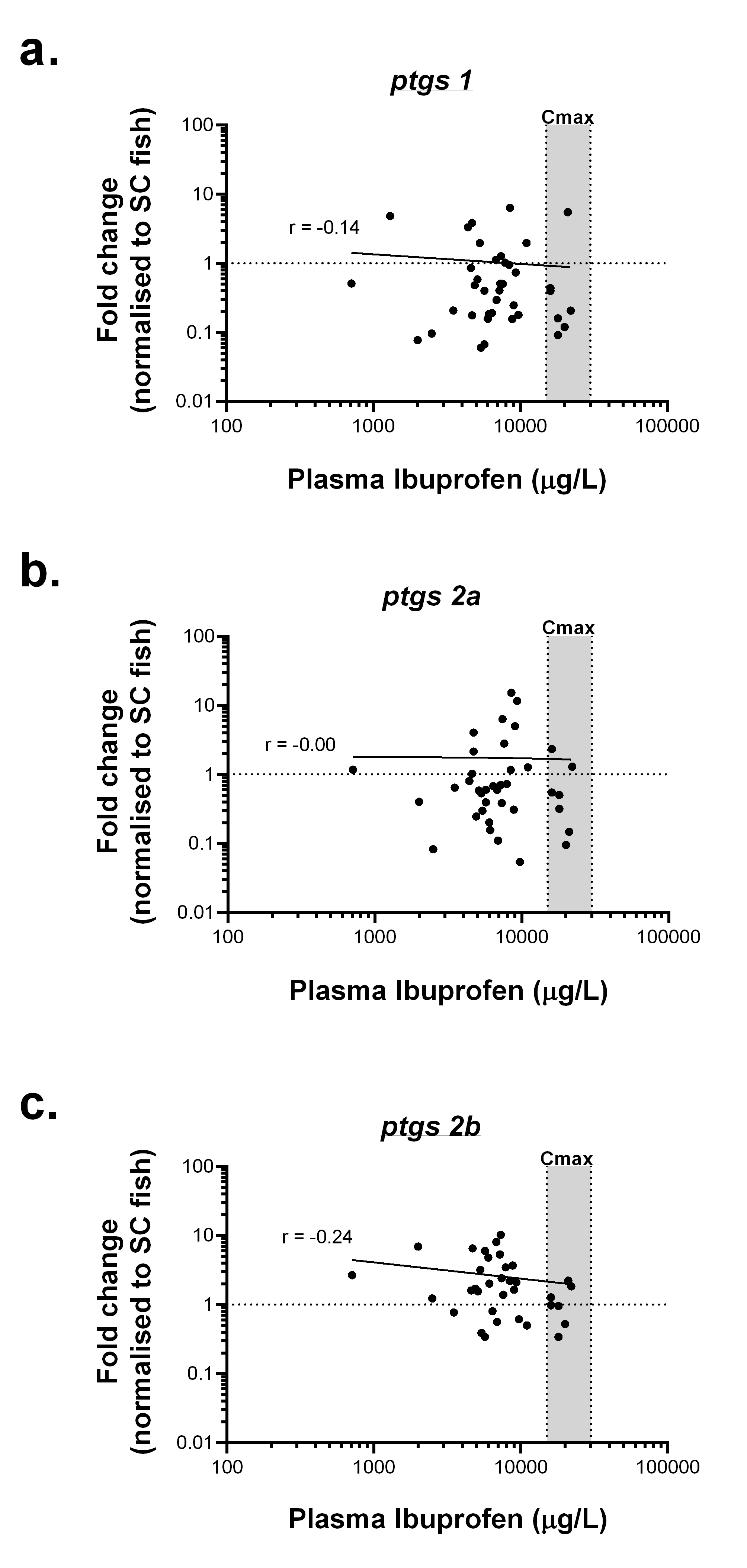
“**Figure S2**. The relationship between *cyclooxygenase* (*ptgs)* gene expression in gills and plasma ibuprofen concentration in fathead minnows (n= 36-40) exposed for 72 h to 370 µg/L ibuprofen. Gene expression is shown as fold change relative to the average of the solvent control (SC) fish (represented as 1). C_max_ = human therapeutic plasma concentrations.”


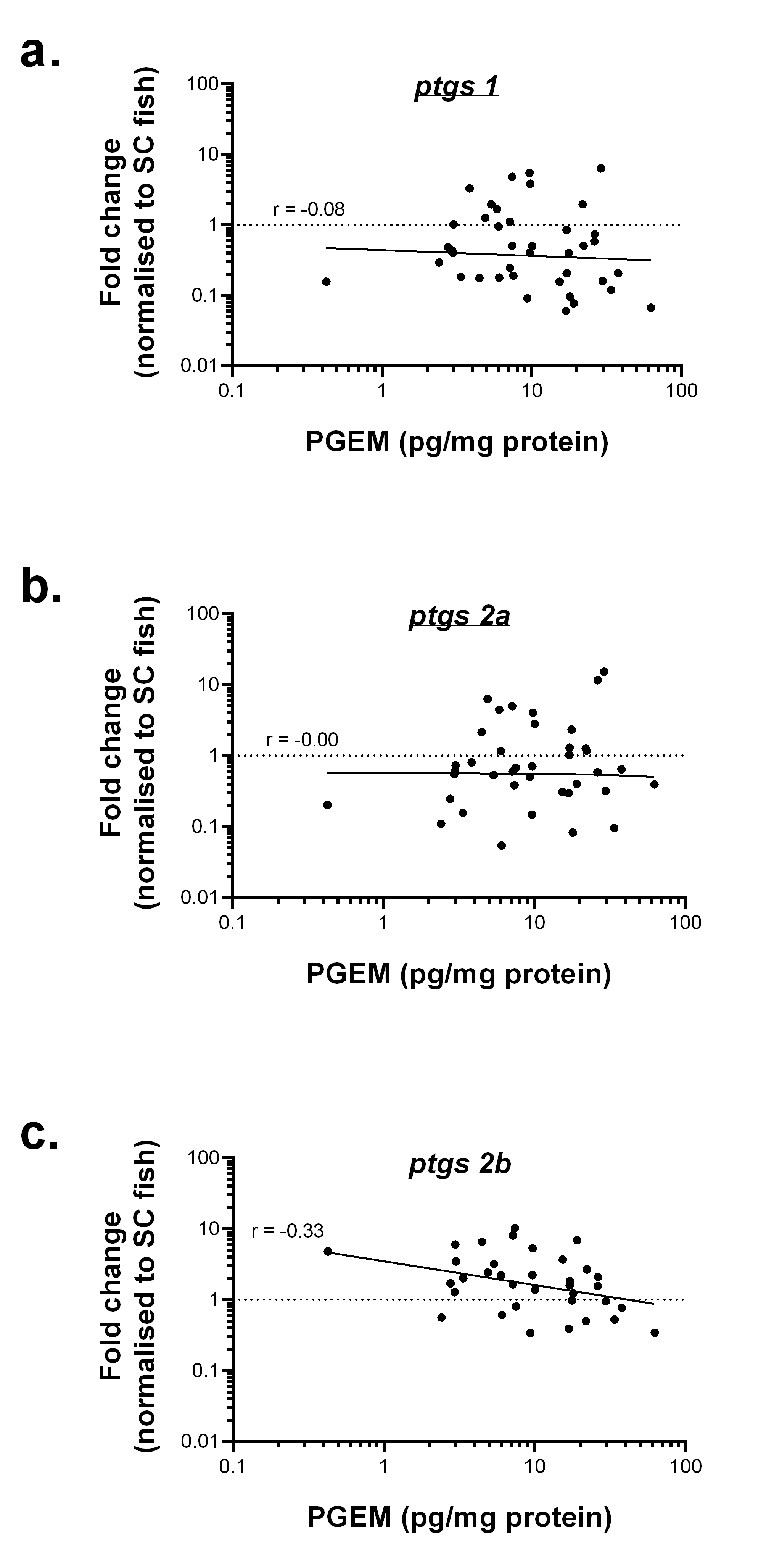
“**Figure S3**. The relationship between *cyclooxygenase* (*ptgs)* gene expression and Prostaglandin E metabolite (PGEM) levels in the gills of fathead minnows exposed (n= 36-40) for 72 h to 370 µg ibuprofen/L. Gene expression is shown as fold change relative to the average of the solvent control (SC) fish (represented as 1).”

**Appendix A**

**Chemical analysis of ibuprofen in water and blood plasma**

*Preparation of standards and samples*: For the quantification of ibuprofen in water samples, calibration standards were prepared from a stock solution of ibuprofen test material (100 mg/L) in HPLC-grade acetonitrile (ACN) by serial dilution using HPLC-grade water (Sigma-Aldrich, Dorset, UK). The water samples (5 mL) collected from exposure tanks were diluted (1:4) with HPLC-grade water and an aliquot (~1 mL) was transferred to an autosampler vial for analysis. Water samples (5 mL) collected from the solvent control (SC) tanks required no dilution and an aliquot (~ 1 mL) was directly transferred to a vial for analysis. Calibration curves (8-point) were prepared by plotting the peak area of ibuprofen against the standard concentration (0.625-160 µg/L), and were generated using linear regression analysis (r^2^ >0.998). The Limit of Quantification (LOQ) was determined by the lowest measured calibration standard (1.25 µg/L) and the Limit of Detection (LOD) in the solvent control (SC) tanks was ≤ 2.5 µg/L.

For quantification of ibuprofen in blood plasma samples, calibration standards were prepared from the stock solution (100 mg/L) by serial dilution using HPLC-grade water and HPLC-grade ACN containing an internal standard, ibuprofen-d_3_ (500 µg/L) (Sigma-Aldrich, Dorset, UK). Plasma samples from control and exposed fish were spiked (1:4) with HPLC-grade acetonitrile containing ibuprofen-d_3_ (80 µg/L). The samples were transferred to a 96-well plate and thoroughly mixed using a plate shaker for 15 minutes at room temperature to facilitate protein precipitation. The plate was centrifuged at 3,220 x g for 30 min at 20 °C to pellet the proteins. The supernatant (up to 100 µL) was transferred to a fresh well and diluted (1:5) with HPLC-grade water to produce an overall dilution x 25. Calibration curves (8-point) were prepared by plotting the peak area of ibuprofen divided by the peak area of the internal standard: ibuprofen-d_3_, against the standard concentration (0.625-160 µg/L) and were generated using linear regression analysis (r^2^ >0.998). Plasma readings that were outside of the calibration range were diluted further and re-analyzed. The LOD of ibuprofen in fish plasma was ≤ 30 µg/L.

*LC-MS/MS*: The concentrations of ibuprofen in water and fish plasma were determined by reversed-phase Liquid Chromatography coupled with tandem Mass Spectrometry (LC-MS/MS). Sample injections (10 µL) were separated using a Dionex Ultimate 3000 instrument using a Gemini® NX C18 column (50 × 2.0 mm, 3 μm, Phenomenex, CA, USA) kept at 50 °C. Elution was performed under gradient conditions. The mobile phase consisted of 0.1% ammonia in water (eluent A) and 0.1% ammonia in methanol (eluent B) delivered at a flow rate of 500 µL/minute. The gradient conditions (over 5 min) were: Time(min)/%(A); 0/90→ 3/0→ 4/0→ 4.1/90→ 5/90. Ibuprofen was detected using an Ion Trap mass spectrometer (LTQ, Thermo Scientific, UK) with heated electrospray ionization in the negative ionization mode. For increased sensitivity, selected reaction monitoring (SRM) was used for the detection of deprotonated ibuprofen (*m/z* 205>161, loss of carbon dioxide) and the internal standard, ibuprofen-d_3_ (*m/z* 208>164). Data were acquired and processed using Xcalibur^TM^ software (Thermo Scientific, UK). Between four and seven QC samples (spiked with a known amount of ibuprofen or ibuprofen-d_3_) were run to check the recovery and the accuracy during sample preparation. The accuracy of the QC samples was determined by the deviation of the measured concentration from the spiked concentration (in percentage) and was within the 15% threshold set.
